# Identification of common signatures in idiopathic pulmonary fibrosis and lung cancer using gene expression modeling

**DOI:** 10.1186/s12885-020-07494-w

**Published:** 2020-10-12

**Authors:** Dong Leng, Jiawen Yi, Maodong Xiang, Hongying Zhao, Yuhui Zhang

**Affiliations:** 1 Clinical Laboratory, Beijing Chao-Yang Hospital, Capital Medical University, Beijing, 100020 China; 2 Department of Respiratory and Critical Care Medicine, Beijing Chao-Yang Hospital, Capital Medical University, No. 8 Gongti South Road, Beijing, 100020 China; 3 Tokyo Institute of Technology, 4259 Nagatsuta-cho, Midori-ku, Yokohama, Kanagawa 226-8503 Japan; 4 Department of Pathology, Beijing Chao-Yang Hospital, Capital Medical University, Beijing, 100020 China

**Keywords:** Idiopathic pulmonary fibrosis, Lung cancer, Gene expression, Data mining, Mutual exclusivity

## Abstract

**Background:**

Idiopathic pulmonary fibrosis (IPF) is associated with an increased risk for lung cancer, but the underlying mechanisms driving malignant transformation remain largely unknown. This study aimed to identify differentially expressed genes (DEGs) distinguishing IPF and lung cancer from healthy individuals and common genes driving the transformation from healthy to IPF and lung cancer.

**Methods:**

The gene expression data for IPF and non-small cell lung cancer (NSCLC) were retrieved from the Gene Expression Omnibus (GEO) database. The DEG signatures were identified via unsupervised two-way clustering (TWC) analysis, supervised support vector machine analysis, dimensional reduction, and mutual exclusivity analysis. Gene enrichment and pathway analyses were performed to identify common signaling pathways. The most significant signature genes in common among IPF and lung cancer were further verified by immunohistochemistry.

**Results:**

The gene expression data from GSE24206 and GSE18842 were merged into a super array dataset comprising 86 patients with lung disorders (17 IPF and 46 NSCLC) and 51 healthy controls and measuring 23,494 unique genes. Seventy-nine signature DEGs were found among IPF and NSCLC. The peroxisome proliferator-activated receptor (PPAR) signaling pathway was the most enriched pathway associated with lung disorders, and matrix metalloproteinase-1 (*MMP-1*) in this pathway was mutually exclusive with several genes in IPF and NSCLC. Subsequent immunohistochemical analysis verified enhanced MMP1 expression in NSCLC associated with IPF.

**Conclusions:**

For the first time, we defined common signature genes for IPF and NSCLC. The mutually exclusive sets of genes were potential drivers for IPF and NSCLC.

## Background

Idiopathic pulmonary fibrosis (IPF) is a chronic, progressive, and usually fatal interstitial lung disease that is characterized by dysfunction and damage of lung epithelial cells and aberrant pulmonary remodeling. After diagnosis, patients usually have a median survival of 3–5 years, and the main cause of death is respiratory failure [1, 2]. Although the exact mechanisms remain largely unknown, it is widely accepted that genetic and environmental factors leading to alveolar epithelial cell injury trigger the repair process and induce the formation of fibroblast foci, ultimately causing pulmonary fibrosis [3]. IPF is considered as a precancerous lung disorder because occasionally patients with IPF have concomitant lung cancer, and patients with IPF have a 3.34-fold greater risk of developing primary lung cancer than the general population [4, 5].

Lung cancer is the most common malignant tumor and the leading cause of cancer deaths worldwide [6]. Non-small cell lung cancer (NSCLC) is the most common type of lung cancer, accounting for about 85% of lung cancer cases, followed by small cell lung cancer (SCLC) with about 13% of cases [6–8]. From a genetic point of view, lung cancer is a highly heterogenous disease with numerous somatic mutations. These driver alterations are capable of abnormally activating downstream signaling pathways and driving tumorigenesis by suppressing apoptosis and promoting cell proliferation, angiogenesis, invasion, and migration [9]. Although lung cancer is considered as a late complication of IPF, the histological types of lung cancer associated with IPF remain uncertain, with controversial results described in the literature [5, 10, 11]. A recent genomic sequencing study demonstrated that IPF and lung cancer have some somatic mutations in common [12], but the genes driving the transformation from IPF to lung cancer are still unknown.

**Fig. 1:**
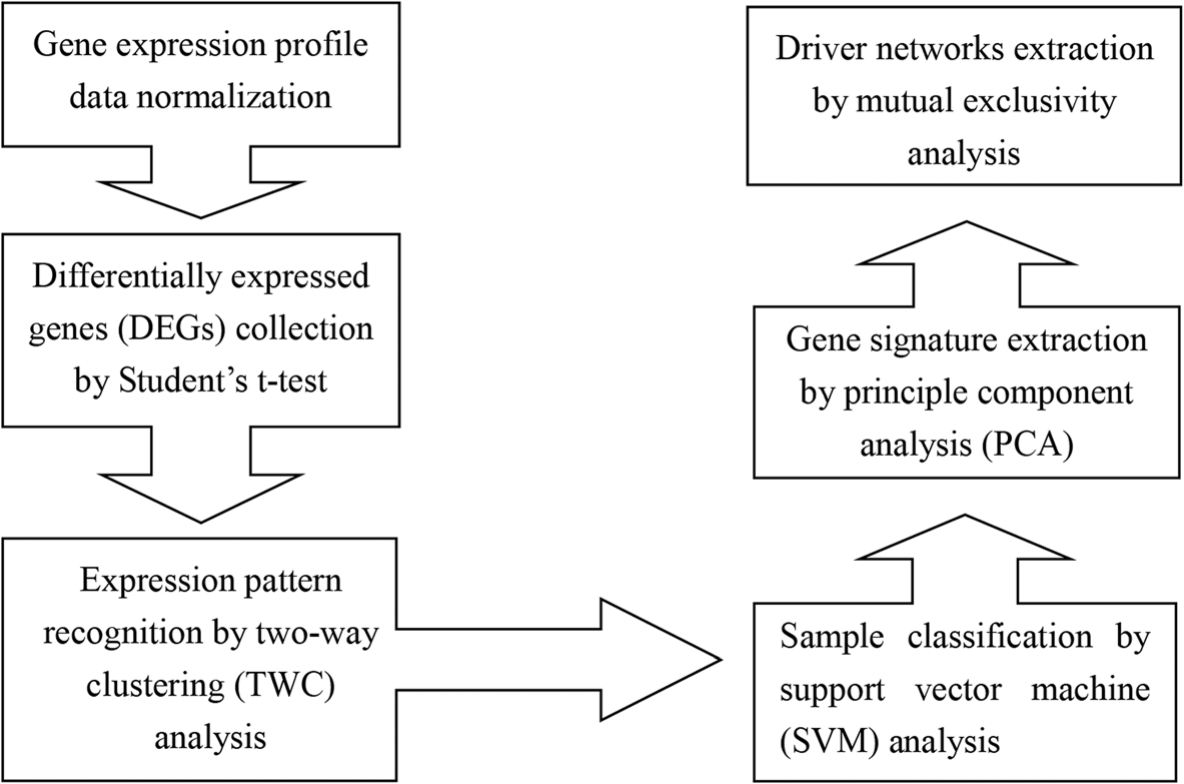
Flow chart of the multistep strategy for the identification of signature genes in this study

In this context, we aimed to identify common genes involved in both IPF and lung cancer by screening the gene expression omnibus (GEO) database for gene expression profile data of lung tissue samples from healthy controls as well as from patients with IPF and NSCLC. A multistep strategy (Fig. 1) was applied to identify differentially expressed genes (DEGs) that distinguish healthy controls from patients with lung disorders, including IPF and NSCLC. In addition, significant gene pairs with mutually exclusive alterations among the lung disorders were screened as potential cancer driver genes. In this research, we identified common signature genes associated with lung disorder development and provided novel pathogenic targets that are relevant for the transformation from IPF to lung cancer.

## Methods

### Gene expression datasets and data preprocessing

The gene expression profile datasets on IPF and NSCLC were searched and downloaded from the GEO repository (http://www.ncbi.nlm.nih.gov/projects/geo/) [13]. The key words for searching were “idiopathic pulmonary fibrosis” and “non-small cell lung carcinoma.” The datasets were filtered to include only the organism of *Homo sapiens*. In this study, the gene expression datasets obtained from the Affymetrix Human Genome U133 Plus 2.0 Array were used as the discovery cohort for data modeling, and the expression datasets obtained from other assays served as the validation cohort. After recognizing the raw data from the IPF and NSCLC patients as well as the healthy controls, probes of the array data were replaced with the corresponding official gene symbols using the GPL570 platform. Duplicates were collapsed using probe medians, as described previously [14, 15]. Log2 transformation was applied to all estimates to make the data more symmetric and plotting easier. CONOR and preprocessCore in the R software package were used to combine the gene expression estimates from different studies [15, 16]. During the data-merging process, the Log2-transformed raw intensity estimates were transformed by iterative clustering until convergence to a minimum sum of the Euclidean distance, which has been proven to be an effective means to remove systematic differences between studies while preserving the completeness of the biological information [17, 18].

### Unsupervised clustering and supervised classification analysis

The unpaired Student’s t-test and two-way clustering (TWC) analysis were used to identify genes that were expressed differentially between disease (IPF and NSCLC) and control samples; genes with *P* < 0.05 were considered as DEGs. During the TWC process, hierarchical clustering algorithms based on the Euclidean distance as a similarity metric and a complete linkage clustering approach were implemented. The hierarchical clustering analysis and visualization were performed using Cluster 3.0 and Java Treeview software. The normalized gene expression data were matched with the DEGs identified by the Student’s t-test to generate the filtered gene expression data with the DEGs, which were subjected to supervised classification via support vector machine (SVM) analysis using multtest and kernlab in the R software package [19] and validated in leave-one-out cross validation (LOOCV) in Python via Anaconda 3.0 software [20]. The SVM analysis uses historical data to predict future events and generates a decision boundary for classification (also known as a hyperplane) that is defined as wx + b = 0, where w is the weight coefficient, x is the input feature vector, and b is the bias. DEGs with a weight coefficient > 1.0 were selected for subsequent analysis. For binary classifications, samples were labeled with unpaired markers and were chosen for model training in each comparison. SVM analysis with a radial basis function kernel was employed to select the optimal separating hyperplane for gene expression classification, in which the variance (σ) and degree of fitting (cross) parameters were set to 0.1 and 5, respectively [21]. The LOOCV procedure used only one sample for each validation, and the rest of the samples were used for training sets in each evaluation to ensure that each sample was validated and to avoid overfitting. The sample size used for each analysis was not too large so that there was not a significant computational burden.

### Dimensionality reduction and signature gene identification

Microarray data possess high-dimensional properties. Each gene may have interactions with others, and any potential interactive relationship is within the data. Principal component analysis (PCA) was utilized to extract key components from the high-dimensional gene expression data. The filtered data with DEGs that were identified by supervised classification analysis (weight coefficient > 1.0) were analyzed with PCA for dimensionality reduction using the prcomp function in the R software. Briefly, the principal components of each DEG were extracted, and the loading coefficients of each gene were calculated, which can be used to assess the ability of the gene to promote or inhibit a disorder (IPF/NSCLC). The standard deviation, proportion of variance, and cumulative proportion are shown in detail in the supplementary data (Summary of principal components). The total number of principal components is equal to the sample number in the data. Those principal components with a cumulative proportion ≥ 85% were analyzed by Bayesian probit regression using the R arm package to evaluate the significance of each principal component. Principal components with *P* < 0.05 were defined as significantly altered. Each principal component in each gene has a corresponding loading coefficient. Genes with a loading coefficient with an absolute value > 0.6 were selected as signature genes.

### Model validation

To validate the models independently, the GSE10667 dataset [22], which was based on the Agilent-014850 Whole Human Genome Microarray 4 × 44 K platform and contained 31 IPF samples and 15 healthy lung samples, was assigned as the validation cohort for gene expression comparison between IPF patients and healthy controls. GSE118370 [23], which was based on the Affymetrix Human Genome U133 Plus 2.0 Array platform and contained 6 pairs of lung adenocarcinoma and normal lung tissue samples, served as the validation cohort for the comparison between NSCLC patients and healthy controls. The IPF/Healthy validation cohort and the NSCLC/Healthy validation cohort were merged with the original IPF/Healthy and NSCLC/Healthy training cohorts, respectively, and clustering analysis was performed on the validation cohorts by TWC. The probabilities of the samples containing IPF or NSCLC were predicted by SVM, based on the original data models. The predictive values were evaluated using receiver operator characteristic (ROC) curves.

### Gene annotation and enrichment analysis

The Database for Annotation, Visualization, and Integrated Discovery (DAVID, v6.8, https://david.ncifcrf.gov/) [24, 25] and the Kyoto Encyclopedia of Genes and Genomes (KEGG) online database (https://www.kegg.jp/) were used to annotate the functions and pathways as well as gene enrichments of the signature genes that were differentially expressed between IPF/NSCLC patients and healthy controls.

### Mutual exclusivity analysis

Synthetic lethality is a type of genetic interaction in which the simultaneous functional loss of two or more genes through mutations, amplifications, or deletions leads to cell death. This genetic phenomenon is vital for cell viability and is emerging as a novel therapeutic target for cancer treatment [26]. Pairs of genes that are altered in a mutually exclusive pattern (i.e., in the opposite direction of gene expression: one upregulated and the other downregulated) in cancers and are often observed in the same pathway are likely to be synthetically lethal. A Markov chain Monte Carlo (MCMC) approach was applied for mutual exclusivity analysis, and the results were visualized via CoMEt, with the marginal probability labeled at the edge of each module [27]. Briefly, a binary alteration matrix was created based on the gene expression profiles (downregulated or upregulated), and mutually exclusive gene modules were produced using the MCMC sampling method. The optimal *k* (number of genes in a mutually exclusive module) and *t* (number of modules) values were determined using the criteria outlined previously [27]; and as a result, *t* = 2 and *k* = 2 were selected for marginal probability modeling (*t* = 3 and *k* = 2 cannot produce perfectly mutually exclusive modules, data not shown). Gene pairs within the mutually exclusive module likely drive disease progression and thus were defined as putative cancer genes [27]. Common putative cancer genes identified in both IPF and lung cancers were predicted to be the driver cancer genes in the transformation from healthy to IPF and lung cancer.

### Patient samples

To validate the signature genes identified by gene expression modeling, the expression pattern of the signature genes showing mutual exclusivity was analyzed in patients diagnosed with IPF or lung cancer. The Ethics Committee of Beijing Chao-Yang Hospital approved the study and waived informed consent (2017-Science-10). Paraffin-embedded lung tissue specimens were obtained from the archives in our hospital from patients with IPF, NSCLC without IPF, and NSCLC associated with IPF for immunohistochemical (IHC) analysis. In addition, tissue specimens obtained from healthy donor or adjacent normal tissue of NSCLC were used as the reference. Table 1 lists the basic clinicopathological features of the patients included in the analysis. The diagnosis was reviewed and confirmed by an independent pathologist.

**Table 1:** Details of the paraffin-embedded lung tissue samples used in this study

Patient	Age at diagnosis	Sex	Diagnosis	Histological type	Clinical Stage	Tissue type	Tissue section identification
1	60	M	NSCLC without IPF	Adenocarcinoma	IIIA	Adjacent normal tissue	a1
2	68	F	NSCLC without IPF	Adenocarcinoma	IA	Adjacent normal tissue	a2
3	Unknown	Unknown	Heathy donor			Normal lung tissue	a3
4	61	M	IPF			Abnormal lung tissue	b1
5	56	M	IPF			Abnormal lung tissue	b2
6	66	M	IPF			Abnormal lung tissue	b3
7	68	F	NSCLC without IPF	Adenocarcinoma	IA	Lung tumor tissue	c1
8	49	M	NSCLC without IPF	Squamous carcinoma	IIIA	Lung tumor tissue	c2
9	60	M	NSCLC without IPF	Adenocarcinoma	IIIA	Lung tumor tissue	c3
10	55	M	NSCLC with IPF	Adenocarcinoma	IA	Lung tumor tissue and adjacent tissue with IPF	d and e

### IHC staining

After deparaffinization with methanol and ethanol and placement in ethylenediaminetetraacetic acid (pH 8.0) for antigen retrieval, the retrieved paraffin-embedded tissue sections were incubated with the polyclonal primary antibody against matrix metalloproteinase-1 (*MMP-1*) (Ab137332, Abcam, Shanghai, China, dilution 1:500) at 4 °C overnight, and then incubated with the secondary antibody for 20 min at room temperature. Finally, diaminobenzidine (DAB: ZSGB-BIO ZLI-9017) staining was performed.

## Results

### Gene expression profile datasets

Three gene expression datasets were identified by the searching criteria as the training cohorts (Table 2). The GSE24206 dataset (USA) [28] contained 17 IPF samples and 6 healthy control samples; IPF diagnosis was based on the multidisciplinary diagnostic criteria described in the American Thoracic Society/European Respiratory Society consensus statement. GSE18842 (Spain) [29] contained 46 NSCLC samples and 45 healthy control samples. GSE43346 (Japan) [30] contained 23 SCLC samples, 42 normal tissue samples from different organisms, and 3 SCLC cell lines. All these training datasets were processed on the Affymetrix Human Genome U133 Plus 2.0 Array platform, which has 54,675 probes representing 23,494 unique genes. As there is an ethnic difference for lung cancer but not for IPF, the GSE24206 (USA) and GSE18842 (Spain) datasets from Caucasians were compared to the GSE43346 (Japan) dataset from Asians; there was no appropriate dataset for SCLC. In this study, we performed genetic analysis only on the datasets GSE24206 and GSE18842, which consist of subjects with the same ethnicity. The genetic analysis was assessed only between IPF and NSCLC.

**Table 2:** Gene expression datasets used as the training cohort

GEO accession No.	Number of samples	Platform	Probes	Genes
GSE24206	IPF patients (*n* = 17)/healthy controls (*n* = 6)	Affymetrix Human Genome U133 Plus 2.0 Array	54,675	23,494
GSE18842	NSCLC patients (*n* = 46)/healthy controls (*n* = 45)	Affymetrix Human Genome U133 Plus 2.0 Array	54,675	23,494

### Raw data reprocessing and super array data

After duplicated measurement collapse and Log2 transformation, gene array data from the three studies containing 23,494 unique genes were obtained. These gene array datasets were normalized and combined to generate a super array dataset, which contained 63 lung disorder (17 IPF and 46 NSCLC) and 51 healthy control lung tissue samples.

### DEGs and gene expression patterns

**Fig. 2:**
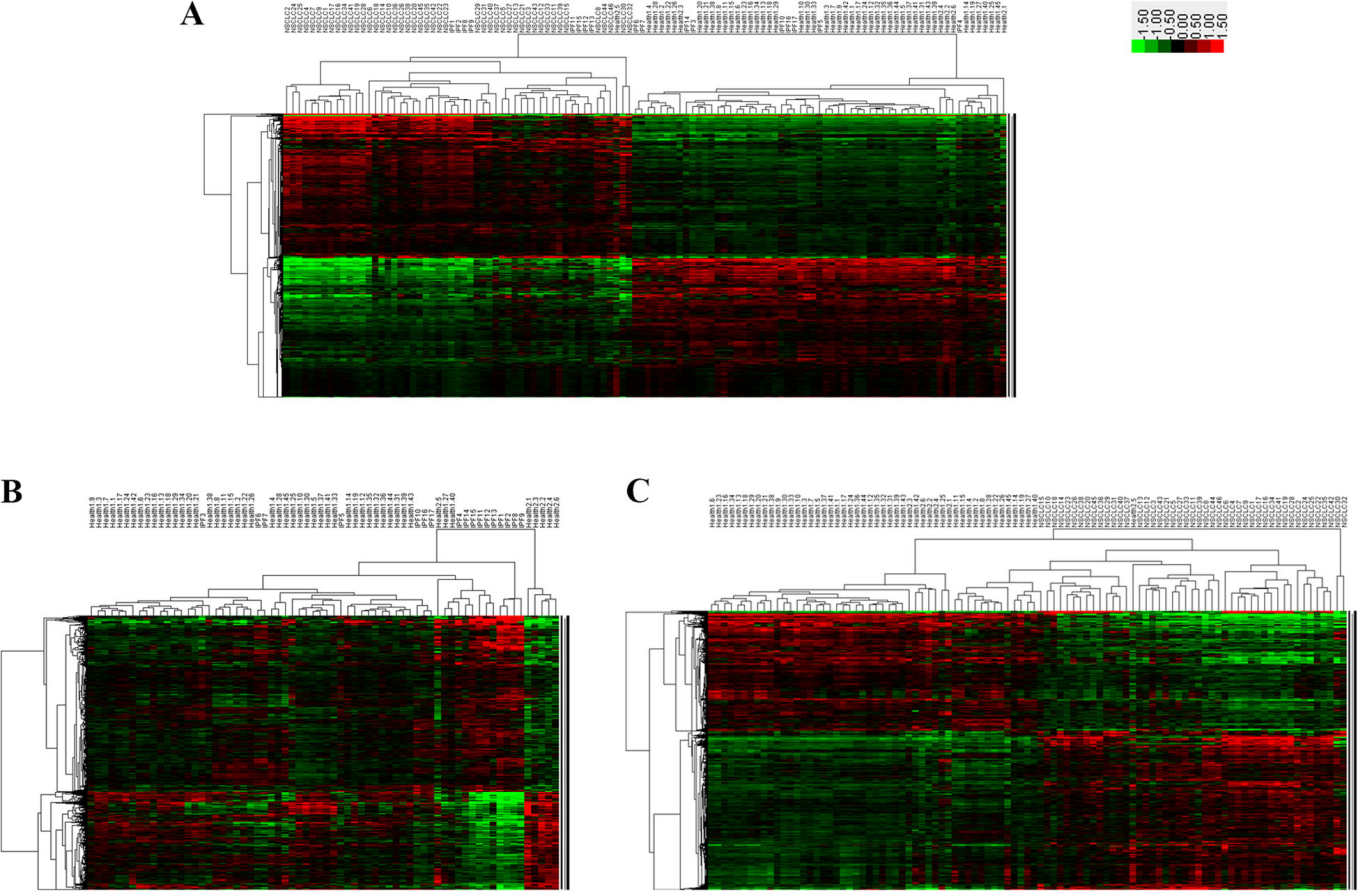
Heat maps of an unsupervised two-way clustering analysis of genes that are differentially expressed between (**a**) patients with lung disorders and healthy controls; (**b**) patients with IPF and healthy controls, and (**c**) patients with NSCLC and healthy controls. The red color indicates upregulation, the green color indicates downregulation, while the black color indicates unchanged expression

**Fig. 3:**
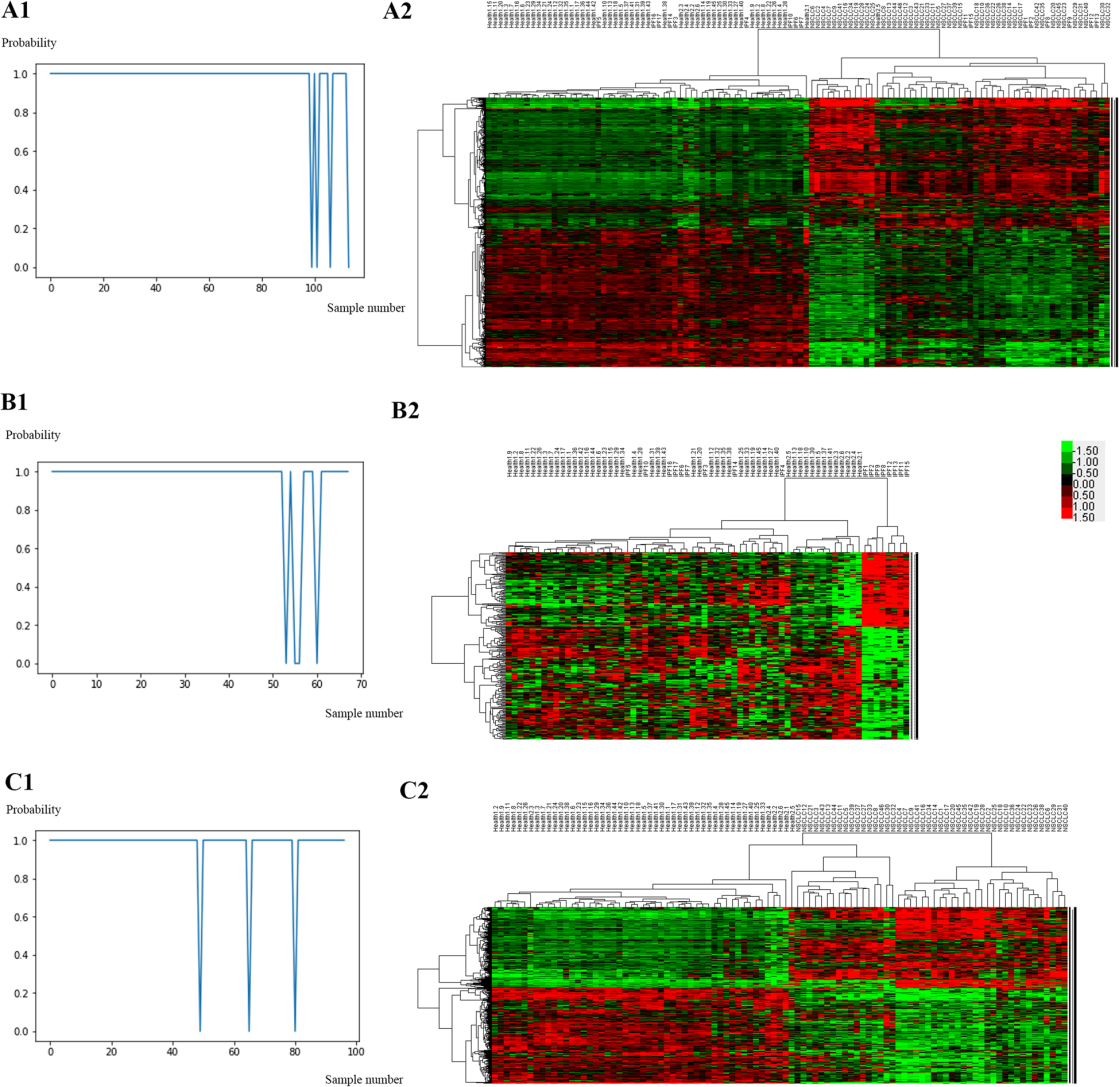
Cross validations and two-way clustering (TWC) analysis of significantly expressed gene data. (A1) Prediction of samples by modeling patients with a lung disorder vs. healthy controls. (A2) Heatmap of TWC analysis of significantly expressed gene data from patients with a lung disorder vs. healthy controls. (B1) Prediction of samples by modeling IPF patients vs. healthy controls. (B2) Heatmap of TWC analysis of significantly expressed gene data from IPF patients vs. healthy controls. (C1) Prediction of samples by modeling NSCLC patients vs. healthy controls. (C2) Heatmap of TWC analysis of significantly expressed gene data from NSCLC patients vs. healthy controls. In panels A1, B1, and C1, the x-axis represents the sample numbers and the y-axis represents the probability of samples expected for each model

The gene expression profiles of patients with lung disorders were distinct from those of the healthy controls, and 11,926 genes were significantly differentially expressed with *P* < 0.05 (Fig. 2). The heat map of clustering analysis revealed three subgroups corresponding to the healthy control, IPF, and NSCLC groups, in sequence, while there were several outliers. Subsequent analysis comparing these subgroups identified 4740 and 10,169 DEGs at *P* < 0.05 from the comparisons of the healthy controls with IPF and NSCLC, respectively. These DEGs identified by unsupervised clustering analysis were trained with SVM analysis to further characterize the gene expression. Sample outliers were picked out by LOOCV (Fig. 3A1 and B1). The SVM model achieved good predictive powers for comparison between patients with lung disorders and healthy controls (91.23%, 104/114), between IPF patients and healthy controls (92.65%, 63/68), and between NSCLC patients and healthy controls (96.91%, 94/97). Figure 3 shows the clustering heat maps of DEGs with weight coefficients > 1.0 after SVM validation; 11,926, 253, and 1021 DEGs with weight coefficients > 1.0 were identified in the comparisons of healthy controls with patients with lung disorders, IPF, and NSCLC, respectively.

### PCA and signature genes

**Fig. 4:**
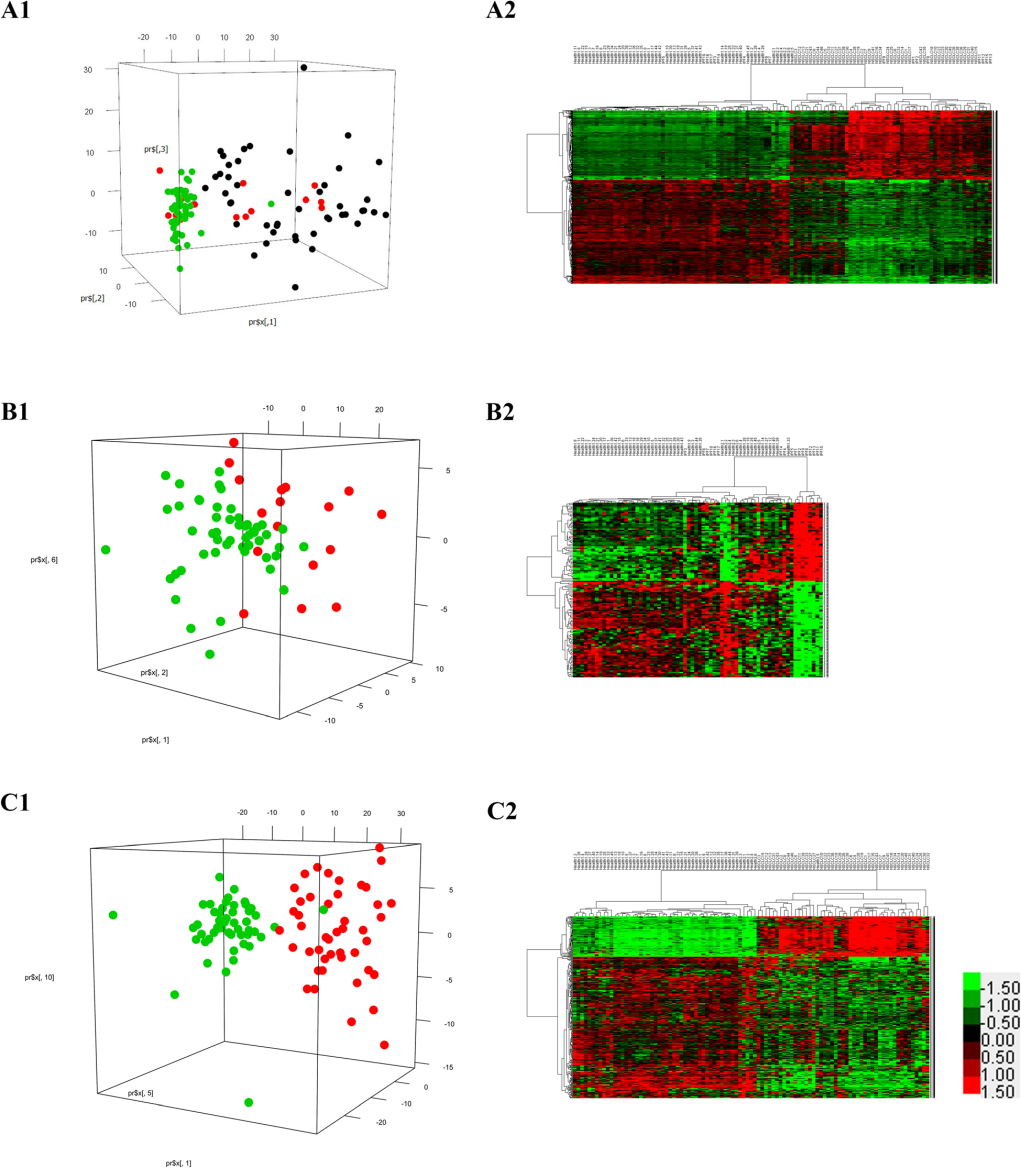
Principal component analysis (PCA) and heat maps of a two-way clustering analysis of signature genes. (A1) PCA separates healthy controls from patients with lung disorders by PC1 (*P* = 0.00217), PC2 (*P* = 0.24174), and PC3 (*P* = 0.76089). (A2) Heat map of signature genes between patients with lung disorders and healthy controls. (B1) PCA separates healthy controls from IPF patients by PC1 (*P* = 0.000282), PC2 (*P* = 0.030919), and PC6 (*P* = 0.002256). (B2) Heat map of 127 signature genes between IPF patients and healthy controls. (C1) PCA separates healthy controls from NSCLC patients by PC1 (*P* = 0.00219), PC5 (*P* = 0.60574), and PC10 (*P* = 0.61893). (C2) Heat map of 396 signature genes between NSCLC patients and healthy controls. In A1, green dots indicate healthy controls, red dots indicate IPF patients, and black dots indicate NSCLC patients. In B1 and C1, green dots indicate healthy controls, and red dots indicate disease samples. In panels A1, B1, and C2, the numbers on each coordinate axis reflect the loading coefficients for genes

PCA was conducted to identify the most significant genes that distinguish healthy subjects from patients with lung disorders. The first principal component (PC1) was found to be the most significant in all comparisons of healthy controls with patients with IPF and NSCLC (the output files of PCA summaries and Bayesian probit regression are shown in the supplementary data). Table 3 shows the significant principal components (*P* < 0.05) and the number of signature genes, which were defined as an absolute loading coefficient > 0.6, for all comparisons. As illustrated in Fig. 4, the top three significant principal components tended to separate healthy controls from patients with lung disorders in all comparisons, and the signature genes successfully distinguished healthy controls from patients with lung disorders. The PCA summaries and Bayesian probit regression results are shown in the supplementary information. We compared the signature genes in all comparisons and identified 79 common signature genes shared by IPF and NSCLC. These common signature genes had the same expression profiles, that is, they were upregulated or downregulated across IPF and NSCLC.

**Table 3:** Principal components significantly related to disease status of the lung and signature genes

	Disease vs. Control	IPF vs. Control	NSCLC vs. Control	
Significant principal components	PC1 (*P* < 0.01)	PC1 (*P* < 0.001)	PC1 (*P* < 0.01)	
		PC2 (*P* < 0.05)		
		PC6 (*P* < 0.01)		
		PC10 (*P* < 0.05)		
Signature genes	666	127	396

**Fig. 5:**
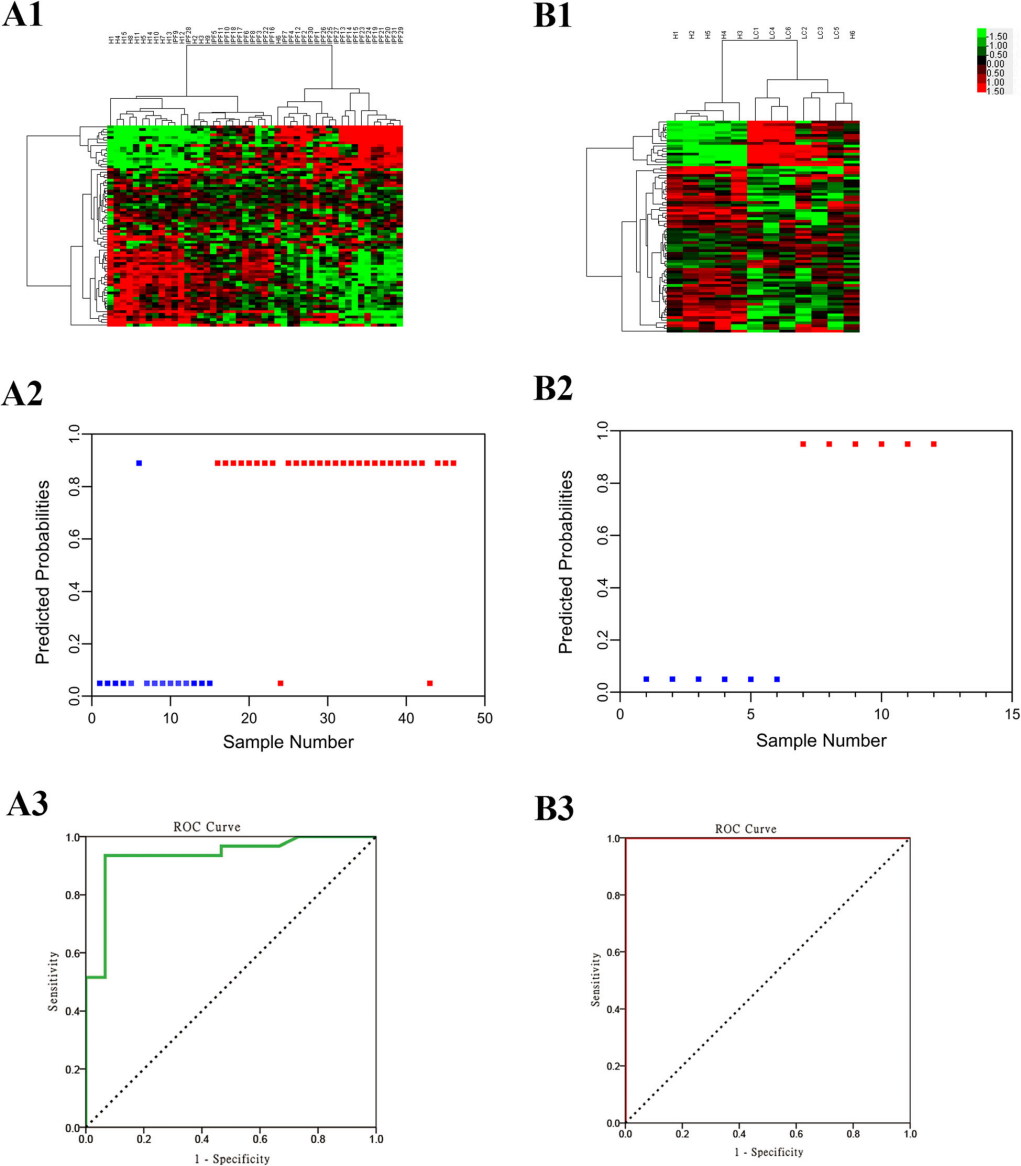
Independent model validation. (A1) Heatmap of TWC analysis of the GSE10667 validation cohort of 31 IPF and 15 healthy control lung tissues by 75 common signature genes. (B1) Heatmap of TWC analysis of the GSE118370 validation cohort of 6 NSCLC and 6 normal control lung tissues by 79 common signature genes. (A2) All validation samples of GSE10667 were assigned by IPF probabilities; the true phenotype of the samples is indicated by the color: 15 blue normal samples and 31 red IPF samples. (B2) All validation samples of GSE118370 were assigned by NSCLC probabilities; the true phenotype of the samples is indicated by the color: 6 blue normal samples and 6 red NSCLC samples. (A3) The ROC curve for the predicted IPF of GSE10667 lung samples by the prior IPF model; the area under the curve was 0.934. (B3) The ROC curve for the predicted NSCLC of GSE118370 lung samples by the prior NSCLC model; the area under the curve was 1.000

Independent validations showed a good classification ability for the signature genes and a good prediction capability of the gene models (Fig. 5). The validation cohort of IPF/Healthy from GSE10667 had 75 common signature genes and a predictive power of 93.48% (43/46) (Fig. 5 A1). The validation cohort of NSCLC/Healthy from GSE118370 had the same 79 common signature genes and a predictive power of 91.67% (11/12) (Fig. 5 B1). The gene expression models produced by SVM also provided good predictive abilities for both the IPF/Healthy and the NSCLC/Healthy cohorts (Fig. 5 A2 and B2). For model validation, the IPF/Healthy training and validation cohorts had 3843 common genes, and the NSCLC/Healthy training and validation cohorts had 10,169 common genes. According to the given information of the true phenotype and the predicted probabilities of a disorder, ROC curves for a disorder of the gene expression models were constructed (Fig. 5 A3 and B3).

### Gene enrichment and pathway analysis

The 79 common signature genes were uploaded to DAVID and KEGG for gene enrichment and pathway analysis. Table 4 summarizes the top 10 remarkable biological terms related to these common signature genes. The most enriched term was “signal,” which accounted for 26 of 79 common signature genes (*P* < 0.001). KEGG pathway analysis showed that the common signature genes were closely related to the peroxisome proliferator-activated receptor (PPAR) signaling pathway and metabolic pathways (Table 5). The PPAR signaling pathway is activated by fatty acids and their derivatives, and it is involved in lipid oxidation and cell proliferation. The metabolic pathways control enzyme-catalyzed reactions within cells, by which living organisms maintain their biological activities. Acyl-CoA dehydrogenase long chain (ACADL), CD36, *lipoprotein lipase* (LPL), and MMP1 were the four common signature genes in the PPAR signaling pathway; and ACADL, amine oxidase, copper containing 3 (AOC3), hydroxysteroid 17-beta dehydrogenase 6 (HSD17B6), and ribonucleotide reductase family member 2 (RRM2) were in the metabolic pathways. In addition, three common signature genes in the pathways concerning cancer were endothelin receptor B (EDNRB), hedgehog-interacting protein (HHIP), and MMP1.

**Table 4:** The top ten remarkable biological annotations from the common signature genes

Category	Term	Count	Fold Enrichment	***P*** value
UP_KEYWORDS	Signal	26	2.29	4.57E-05
UP_KEYWORDS	Disulfide bond	12	2.75	0.0034
KEGG_PATHWAY	ptr03320: PPAR signaling pathway	4	12.20	0.0038
GOTERM_CC_DIRECT	GO:0005615 ~ extracellular space	11	2.89	0.0038
GOTERM_BP_DIRECT	GO:0070062 ~ extracellular exosome	19	1.91	0.0060
GOTERM_BP_DIRECT	GO:0002803 ~ positive regulation of antibacterial	2	141.37	0.014
GOTERM_BP_DIRECT	GO: 0050848, regulation of calcium-mediated signaling	2	106.03	0.019
UP_KEYWORDS	Protease	5	4.83	0.019
INTERPRO	IPR012848: Propeptide, peptidase A1	2	97.79	0.020
GOTERM_CC_DIRECT	GO:0044295 ~ axonal growth cone	2	90.28	0.022

**Table 5:** The top ten enriched biological pathways

Biological pathway	Genes
PPAR signaling pathway	ACADL, CD36, LPL, MMP1
Metabolic pathways	ACADL, AOC3, HSD17B6, RRM2
Pathways in cancer	EDNRB, HHIP, MMP1
Alzheimer’s disease	LPL, MME
Proximal tubule bicarbonate reclamation	CA2, CA4
Cholesterol metabolism	CD36, LPL
Phagosome	CD36, SFTPD
Relaxin signaling pathway	EDNRB, MMP1
Adrenergic signaling in cardiomyocytes	SCN7A, TNNC1
Chemokine signaling pathway	CXCL14, CXCR2

### Mutually exclusive gene modules

**Fig. 6:**
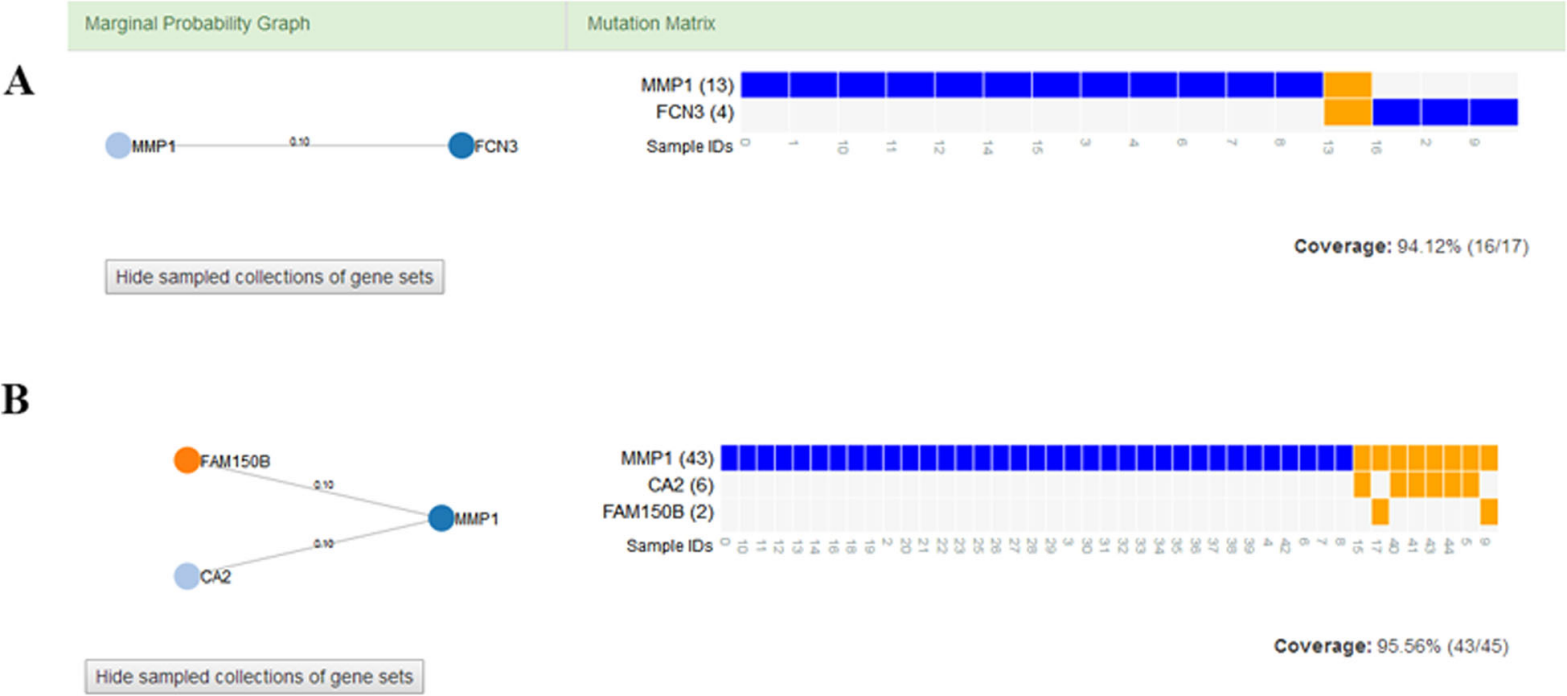
Mutually exclusive gene module of MMP1 in the comparisons between (**a**) IPF patients and healthy controls and (**b**) NSCLC patients and healthy controls. Each dot represents a gene in a mutually exclusive module, and black lines indicate marginal probabilities (Left). Blue rectangles indicate perfectly mutually exclusive samples. Orange rectangles indicate non-mutually exclusive samples

The MCMC method was used to identify mutually exclusive modules in the comparison of healthy controls with IPF and NSCLC. When the edge weight was set to δ = 0.2, three different types of cliques were generated for these comparisons; while when δ = 0.1, more mutually exclusive modules were obtained for each comparison (data not shown). Seven cliques involving 33 genes and 11 cliques involving 39 genes were generated in the comparisons of healthy controls with IPF and NSCLC, respectively. Among these genes, MMP1, PPAP2C, SFTA1P, and LPL were found to be shared between IPF and NSCLC. MMP1 was mutually exclusive in the two comparisons and had a high coverage power (Fig. 6). MMP1 was mutually exclusive with FCN3 in IPF, and with FAM150B and CA2 in NSCLC. SFTA1P had a relatively low coverage power (9.89%, 4/45) in the comparison between NSCLC and healthy controls.

### IHC analysis of MMP1

**Fig. 7:**
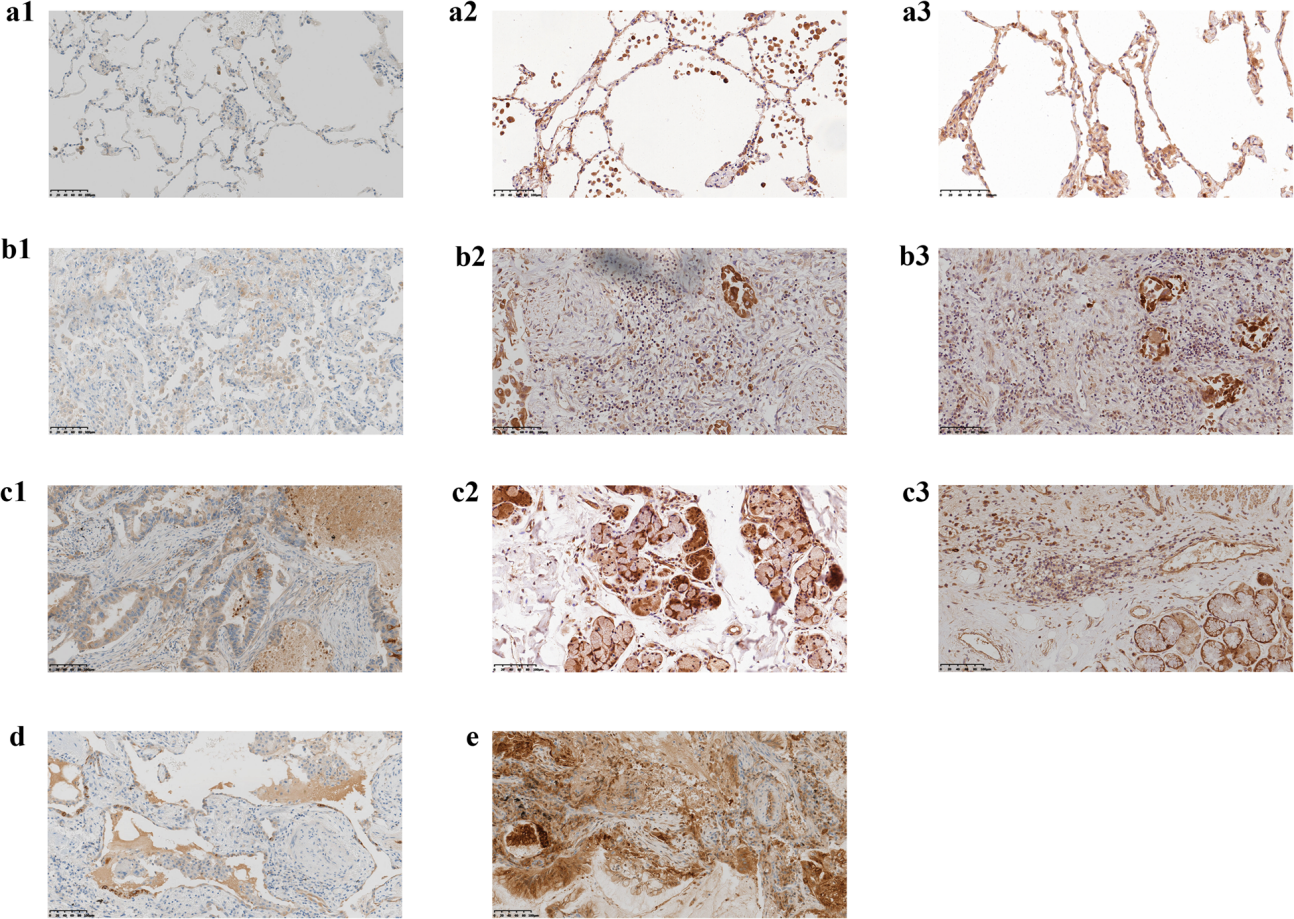
Immunohistochemical analysis of MMP1 (**a**) and computed tomography (CT) scanning images of a patient with IPF (**b**). DAB staining (brown color) indicates positive MMP1 expression in adjacent normal lung tissue of NSCLC without IPF or healthy normal tissue (a1-a3), IPF tissue (b1-b3), lung tumor tissue of NSCLC without IPF (c1-c3), IPF tissue of NSCLC with IPF (**d**), and tumor tissue of NSCLC with IPF (**e**). The IPF sample (b1-b3) is more severe than the IPF-progressed NSCLC sample from a patient who had not been diagnosed with lung cancer (**d**) and is also more severe than the IPF-progressed NSCLC sample from a patient who was diagnosed with lung cancer (a1-a2)

The gene expression of MMP1 was further validated in lung tissue samples from IPF patients, NSCLC patients without IPF, and NSCLC patients with IPF by IHC staining. Positive IHC staining of MMP1 was observed in all of the tested samples, but the staining intensities were different. A slightly increased expression of MMP1 was observed in the NSCLC-without-IPF tumor tissue and the IPF tissue compared with the adjacent normal lung tissue sections or with the healthy donor lung tissue. MMP1 expression was mostly observed in the alveolar epithelium in the IPF tissue; whereas in the NSCLC-without-IPF tumor tissue, MMP1 presented predominantly in the glandular epithelium. The NSCLC-with-IPF tumor tissue displayed a distinctly stronger expression of MMP1 compared with the other tissue samples, and the staining was mainly localized to the glandular epithelium and extracellular stroma. Specifically, MMP1 staining was more intense in the tumor section of NSCLC with IPF compared with the paired IPF section, and compared with the tumor section of NSCLC without IPF. Particularly, there was no association between MMP1 expression and tumor staging, but MMP1 expression was probably correlated with the severity of IPF (Fig. 7).

## Discussion

Previous studies have indicated that patients with IPF are at high risk for lung cancer [10, 31, 32], and the survival of patients with IPF is clearly related to the development of lung cancer [33, 34]. The association between IPF and lung cancer also has been demonstrated by the clinicopathological and imaging characteristics of lung cancer associated with IPF [35, 36]. These findings suggest the need for more research to elucidate the underlying mechanisms related to malignant transformation from IPF to lung cancer, which will provide insight into lung tumorigenesis and unveil therapeutic targets for lung disorders. Previous genetic studies have reported genetic alterations associated with IPF, lung cancer, and lung cancer associated with IPF using gene expression microarray or targeted next-generation sequencing assays [12, 28–30]. Specific gene signatures for both IPF and lung cancer were observed in the corresponding lung tissues, and the findings of germline mutations in lung cancer associated with IPF revealed the existence of a genetic predisposition to lung cancer in patients with IPF, which is different from the well-accepted mechanism of lung tumorigenesis induced by chronic pulmonary damage triggered by environmental stimuli, such as cigarette smoking [12]. Cigarette smoking is a well-recognized risk factor for the development of IPF and lung cancer. However, due to the lack of smoking data in the gene expression profile datasets, we were unable to control for its confounding effects. Further studies are necessary to validate the findings of our study after controlling for potential confounders. Yet, the exact molecular mechanism of malignant transformation from IPF to lung cancer is still unclear. Our results showed for the first time that IPF shares genetic alterations, especially signature cancer genes, in common with NSCLC.

In this study, we examined the gene expression profile data of IPF and NSCLC from the GEO repository using bioinformatic analysis. MMP1 was identified as a promising cancer driver gene related to the transformation from healthy cells to IPF, and from healthy cells to NSCLC. MMP1 expression was further validated in IPF and NSCLC tissues by IHC, and the results could partly confirm the enhanced expression of MMP1 in NSCLC associated with IPF. Studies with more tissue samples are needed to quantitatively confirm these findings. Interestingly, there were no differentially expressed genes between IPF and NSCLC samples (data not shown). This result partially confirms the well-recognized opinion that from the perspective of genetic alteration, IPF shares common hallmarks in response to fundamental pathogenic events like cell proliferation, myofibroblast origin, etc. [4, 37]. To identify gene signatures for lung disorders, the gene expression profiles of IPF and NSCLC were compared with those of healthy controls individually, and 79 common signature genes were found to have differential expression shared by these lung disorders, supporting previous findings that IPF shares oncogenic pathways in common with cancer [38, 39].

In this study, the gene expression profile data from IPF and NSCLC patients as well as healthy controls were pooled and clustered to visualize the similarities and differences in their expression. The gene expression patterns associated with these disorders were gradually filtered through unsupervised and supervised classification and dimensional reduction; distinct expression patterns were observed. The SVM classifier and PCA used in this study are novel tools for biological data mining and have been successfully applied previously for lung disorder classification [40, 41]. The process of SVM learning is a process of machine learning that can predict future data from modeling past data. The SVM classifier can predict the possibility of one sample in each group. Theoretically, any gene with certain gene expression estimates will have some ability of classification from one group to another, and there is no specific threshold for the weights. The cut-off of 1.0 was a random choice for our study. Similarly, PCA extracts principal components while working out the loading coefficients for each gene. The cut-off of 0.6 for the loading coefficient was also a random choice for this study. Taken together, the gene expression patterns predicted by SVM classification are believed to have potential applications for the early diagnosis and prognostic prediction for specific treatment regimens. Although independent validations with quantitative evaluations have shown good predictive abilities of the models, it should be noted that the gene expression patterns identified in this study should be optimized by more samples in further studies.

It is reasonable to hypothesize that the common signature genes shared by IPF and lung cancer are gene signatures for lung cancer associated with IPF and that signaling pathways enriched by these genes may be involved in disease progression. In this study, the most enriched pathway was the PPAR signaling pathway, which is involved in cell proliferation. Although the exact mechanism of PPARs in lung fibrosis and lung cancer is largely unknown, a PPARγ agonist has been found to exert antitumorigenic effects in both IPF and lung cancer by inhibiting myofibroblast differentiation and activating phosphatase and tensin homolog (PTEN) [42, 43], consistent with our study, highlighting the importance of the PPAR pathway in lung cancer associated with IPF. ACADL, CD36, LPL, and MMP1 were the signature genes in the PPAR pathway that were identified to be shared among IPF and lung cancer. ACADL, downregulated in this study, has been reported to induce pulmonary surfactant dysfunction [44] and to be a core signature gene that differentiates NSCLC from normal tissue [45]. CD36, which was downregulated in this study, is a sensor of diacylglycerides, usually acts as a receptor to bind with a broad range of ligands, and has been reported to be downregulated in SCLC cell lines [46]. LPL, which was downregulated in this study, was found to have decreased expression but increased activity in lung cancer tissue compared with adjacent noncancer lung tissue [47]. MMP1 was the most significantly altered common signature gene shared across IPF and lung cancer. It was originally reported to be overexpressed and associated with the early onset of lung cancer [48]. In addition, increased MMP1 plasma concentrations have been observed in patients with IPF [49]. Notably, MMP1 also was assigned to the “pathways in cancer” from the KEGG database. EDNRB and HHIP in this pathway were found to be downregulated in this study, which is consistent with previous reports regarding patients with lung cancer [50, 51].

In this study, we identified four common cancer genes that were shared in common between IPF and NSCLC and were mutually exclusive, while each of these genes had their own mutually exclusive partners for each lung disorder. It is generally accepted that genetic alterations that drive cancer are often mutually exclusive [52]. Therefore, it is theoretically reasonable to propose that driver genes in the malignant transformation from IPF to NSCLC could probably be derived from mutually exclusive genes shared in common by IPF and NSCLC. Among the four mutually exclusive genes identified in this study, PPAP2C and LPL were significant in NSCLC, suggesting their involvement in lung cancer but not in IPF. On the other hand, MMP1 had its unique mutually exclusive partners in IPF and NSCLC, respectively, and had high coverages for all these lung disorder types. In addition, MMP1 demonstrated the highest fold change among the 79 common signature genes. Subsequent IHC analysis revealed that MMP1 expression was stronger in the cancer tissue of the patient with stage IA NSCLC associated with IPF than that of the patient with stage IIIA NSCLC without IPF, suggesting a greater contribution of MMP1 to the derivation of IPF than to the progression of NSCLC. Due to low availability of human lung tissues in the clinic, this observation needs to be further investigated in more samples and quantitative experiments. Regardless, this observation is somewhat in agreement with previous findings that insertion of a guanine (G) at nucleotide position 1607 (rs11292517) in the promotor region of MMP1 results in a 2G allele that is a susceptible factor for IPF and lung cancer [48, 53–56]. Previous investigations have shown that MMP1 participates in the onset of IPF through extracellular matrix remodeling, basement-membrane breakdown, epithelial cell apoptosis, cell migration, and angiogenesis. MMP1 also has been reported to promote tumor invasion and metastasis via loosening cell adhesion. As such, MMP1 is likely to be a candidate target gene that drives malignant transformation from IPF to lung cancer.

The outliers observed during the analysis were probably due in part to the epigenetic influence of DNA methylation, histone tail modification, and noncoding RNAs, as suggested previously, which are influenced by other diseases, aging, smoking, diet, and other environmental stimuli [57]. Because these factors were not well defined in the gene expression profile data included in this study, we were unable to stratify the samples by these factors. A tendentious pattern of clustering from healthy controls to IPF and NSCLC in terms of the gene expression profiles was observed in this study. This finding is consistent with previous investigations that supported the transformation from IPF to lung cancer [58–60]. Unfortunately, the gene expression profile data of lung cancer associated with IPF were not available to verify whether the tendentious transformation from normal healthy tissue to IPF, and then to lung cancer holds. Because patients with lung cancer combined with IPF are at high risk for surgery, it was difficult to obtain resected lung tissue for immunohistochemistry analysis. Therefore, the current results should be considered as preliminary. Further studies based on abundant lung tissues would give a more precise expression tendency of signature genes shared by IPF and NSCLC.

## Conclusions

To the best of our knowledge, this is the first study to compare the gene expression profiles across healthy, IPF, and NSCLC samples using bioinformatic analysis based on published gene expression data. We identified signature genes common to IPF and lung cancer as well as the common signaling pathways involved in tumor development. By introducing mutually exclusive expression analysis, we found potential driving modules for both IPF and NSCLC. Of these genes, MMP1 appeared to be the most promising driver gene showing significant differential expression in the transformation from healthy to IPF and NSCLC, supporting its potential as a novel therapeutic target for IPF, lung cancer, and IPF-progressed lung cancer. Further investigations to verify the gene expression patterns identified in this study in more samples with quantitative experiments and to explore the underlying mechanism in primary cells from IPF, lung cancer, and IPF-associated lung cancer are needed to provide new insight into lung tumorigenesis and targeted therapy.

## Supplementary information

**Additional file 1.** PCA summaries for Healthy vs. Disorder, Healthy vs. IPF, and Healthy vs. NSCLC. Bayesian probit regression results for Healthy vs. Disorder, Healthy vs. IPF, and Healthy vs. NSCLC.

## Data Availability

All relevant data are available upon request from the corresponding author.

## Abbreviations

ACADL: Acyl-coA dehydrogenase long chain
AOC3: Amine oxidase, copper containing 3
DAB: Diaminobenzidine
DAVID: Database for annotation, visualization, and integrated discovery
DEGs: Differentially expressed genes
EDNRB: Endothelin receptor B
GEO: Gene expression omnibus
HHIP: Hedgehog-interacting protein
HSD17B6: Hydroxysteroid 17-beta dehydrogenase 6
IHC: Immunohistochemical
IPF: Idiopathic pulmonary fibrosis
LOOCV: Leave one out cross validation
LPL: Lipoprotein lipase
MCMC: Markov chain Monte Carlo
MMP1: Matrix metalloproteinase-1
NSCLC: Non-small cell lung cancer
PCA: Principal component analysis
PPAR: Peroxisome proliferator-activated receptor
ROC: Receiver operator characteristic
RRM2: Ribonucleotide reductase family member 2
SCLC: Small cell lung cancer
SVM: Support vector machine
TWC: Two-way clustering
